# Emerging Modes of Treatment of IgA Nephropathy

**DOI:** 10.3390/ijms21239064

**Published:** 2020-11-28

**Authors:** Dita Maixnerova, Vladimir Tesar

**Affiliations:** 1st Faculty of Medicine, General University Hospital, Department of Nephrology, Charles University, 128 08 Prague, Czech Republic; vladimir.tesar@vfn.cz

**Keywords:** IgAN, proteinuria, CKD, progression, ACEI, corticosteroids

## Abstract

IgA nephropathy is the most common primary glomerulonephritis with potentially serious outcome leading to end stage renal disease in 30 to 50% of patients within 20 to 30 years. Renal biopsy, which might be associated with risks of complications (bleeding and others), still remains the only reliable diagnostic tool for IgA nephropathy. Therefore, the search for non-invasive diagnostic and prognostic markers for detection of subclinical types of IgA nephropathy, evaluation of disease activity, and assessment of treatment effectiveness, is of utmost importance. In this review, we summarize treatment options for patients with IgA nephropathy including the drugs currently under evaluation in randomized control trials. An early initiation of immunosupressive regimens in patients with IgA nephropathy at risk of progression should result in the slowing down of the progression of renal function to end stage renal disease.

## 1. Introduction, Diagnosis, Pathogenesis

IgA nephropathy (IgAN) is the most common primary glomerulonephritis worldwide with potentially serious renal outcome leading, in 30–50% of patients, to end stage renal disease (ESRD) within 20 to 30 years of follow-up [1,2].

Diagnosis of IgAN is currently based on evaluation of renal biopsy specimens with the demonstration of mesangial IgA1-dominant or co-dominant immunodeposits [3,4].

Clinical risk factors predicting poor renal outcome in patients with IgAN include time-averaged proteinuria, hypertension, decreased estimated glomerular filtration rate (eGFR) [2,5] at presentation or during follow up as well as histological findings evaluated using C-MEST classification [6,7].

IgAN is an autoimmune disease arising from a multi-hit pathophysiological process and is believed to be caused by the interaction of genetic and environmental contributing factors [2,8,9]. Genome-wide associated studies indicated a pathogenetic role of the intestinal immunity abnormalities in IgAN and confirmed a direct link of IgAN with the risk of inflammatory bowel disease or maintenance of the intestinal epithelial barrier [9].

A key role in the pathogenesis of IgAN is played by aberrantly glycosylated forms of IgA1 with galactose-deficient *O*-glycans (galactose-deficient IgA1; Gd-IgA1), which are recognized by antiglycan autoantibodies of IgG and/or IgA1 isotype, resulting in the formation of circulating immune complexes [10,11]. These complexes are deposited in the glomerular mesangium with subsequent mesangial-cell activation and local stimulation of the complement system, proliferation of mesangial cells, and production of extracellular matrix and cytokines, which could alter podocyte gene expression and glomerular permeability in clinical presentation of proteinuria and tubulointerstitial changes in IgAN [12,13,14,15]. If unabated, the damage progresses to glomerulosclerosis and interstitial fibrosis with impaired renal function with subsequent end-stage renal disease.

## 2. Treatment

Which goals do we have in treatment of patients with IgAN? We have to respect the possibility of induction of clinical remmission, reduction in proteinuria and hematuria, stabilization of renal parameters without further decline of GFR, a decrease in the rate of progression, prevention of the necessity of renal replacement therapy as well as the risk of adverse events in patients with corticosteroids and other immunosuppressive regimens. The balance of risks and benefits needs to be taken into account in all individual cases.

### 2.1. Low Risk Patients with IgAN

Low risk patients with minor urinary abnormalities (proteinuria ˂ 0.5 g/d and/or isolated microhematuria), normal glomerular filtration rate (GFR), no hypertension and without histological activity are at low risk of progression, do not require treatment and should be checked annually for at least 10 years according to KDIGO guidelines (Table 1) [16]. Supportive management such as diet modification, weight optimisation and smoking cessation should be taken into consideration. The ideal treatment of the initial phase of IgAN is focused on the decrease in or inhibition of production of Gd-IgA1 by means of a low cost drug with minimal adverse effects. Intestinal-associated lymphoid tissue and mucosal immunity show attractive targets [17,18]. Intestinal microbiota and/or diet regimens including gluten-free diet were suggested in an experimental mice model and human pilot studies [17,19].

Low protein diets were reported to decrease renal function decline [20]. The restriction of sodium was associated with sodium sensitivity of blood pressure and correlated with renal ultrastructural damage [21]. The decrease in proteinuria was shown even in normotensive patients with IgAN due to low-sodium diets [21]. The damaging effects of heightened sodium sensitivity are mediated due to the renin-angiotensin system and it was confirmed that sodium restriction improves the antiproteinuric effects of RAS inhibition in patients with IgAN [22]. Increased morbidity and mortality in patients with chronic kidney disease were associated with extreme body mass index [23]. The relation between body mass index and the probability of end stage renal disease was shown in patients with IgAN in a Chinese study [23]. Increased body mass index associated with lower remission of proteinuria subsequent to treatment was confirmed in a Japanese study [24]. It was assumed that obesity increased proteinuria in connection with hypertension and other parts of metabolic syndrome. Moreover, the advantages of losing weight in overweight patients with IgAN through protein/sodium restriction, and by attaining maximal control of hypertension using treatment with inhibitors of the renin-angiotension system, were confirmed [25].

In addition, the benefits of quitting smoking on slowing down the progression of renal function decline in patients with IgAN were assessed [26].

### 2.2. Intermediate Risk Patients with IgAN

Intermediate risk IgAN patients with proteinuria > 0.5–1 g/d, and/or hypertension and a reduced GFR (without active histological findings in renal specimens) should obtain optimized supportive treatment with the inhibitors of the renin-angiotensin system (RAS) with up-titration of the drug depending on blood pressure to achieve proteinuria < 1 g/d, and should be thoroughly monitored [16]. The suggested therapeutic goals of blood pressure in patients with proteinuria < 1 g/d are <130/80 mmHg, and <125/75 mmHg in patients with initial proteinuria >1 g/d (Table 1) [16]. RAS inhibition was demonstrated to reduce proteinuria and may possibly also reduce the progression of chronic kidney disease in patients with IgAN; at least part of this effect is probably mediated by improved control of blood pressure. High risk of mortality of patients with IgAN might be caused by chronic kidney disease [27,28,29].

It was suggested that patients with persistent proteinuria ≥ 1 g/d, despite 3–6 months of optimized supportive care (including RAS and blood pressure control), and GFR > 50 mL/min per 1.73 m^2^, should receive a 6-month course of corticosteroid therapy (Table 1) [16]. The 10-year renal survival and median proteinuria were significantly better in patients who received a 6-month regimen of corticosteroids compared to patients with a symptomatic treatment (97% vs. 53%, *p* = 0.0003; median proteinuria 1.9 g/24 h at baseline, 1.1 g/24 h after six months and 0.6 g/24 h after a median of seven years) [30]. Other randomized and observational trials supported the potential effect of corticosteroids in IgAN [27,31]. The retrospective analysis of the VALIGA study showed, in patients treated with steroids and RAS blockers (RASB), significant reduction in proteinuria, renal function decline and increased renal survival, not only in patients with normal renal function but also in patients with eGFR ˂ 50 mL/min per 1.73 m^2^ (reaching proteinuria <1 g/day in 74% patients with corticosteroids and RASB vs. 37% in patients with RASB; slope of eGFR −0.3 ± 6.2 mL/min/1.73 m^2^/year in patients with corticosteroids and RASB vs. −4.8 ± 7.4 mL/min/1.73 m^2^/year in patients with RASB; *p* = 0.001) [32]. Nevertheless, little information is available about the doses, the duration of steroid treatment and the adverse events caused by corticosteroids [27].

The TESTING study, a randomized study by Lv et al., included 262 patients with persistent proteinuria 1 g/day and estimated GFR 20–120 mL/min/1.73 m^2^, who were randomly assigned to receive 0.6–0.8 mg/kg/day of oral methylprednisolone or matching placebo [33]. The temporary results demonstrated a reduction in time-averaged proteinuria and a decreased rate of progression of CKD in the steroid-treated arm (1.37 vs. 2.36 g/day (42% lower), *p* < 0.01; −1.7 vs. −6.8 mL/min/1.73 m^2^/year, *p* = 0.031) [33]. However, after a median follow-up of 1.5 years, serious adverse events occurred in patients with corticosteroids vs. placebo groups (14.7% vs. 3.2%, HR 4.95 (95% CI 1.87–17.0), *p* = 0.03) [33]. The additional long-term follow-up might reveal the balance of risks and benefits of steroid treatment [33]. Multiethnic trials such as the ongoing TESTING Low Dose trial (NCT01560052), should evaluate this issue further [34].

The recent STOP-IgAN Clinical Trial showed that the addition of immunosuppressive therapy (corticosteroids and cyclophosphamide followed by azathioprine) to intensive supportive care (ACEI-inhibitors or ARB) in patients with high-risk IgA nephropathy induced full remission of proteinuria (OR 4.82 (95% CI 1.43–16.30), *p* = 0.01) but did not significantly improve renal function (OR O.89 (95% CI 0.44–1.81), *p* = 0.75) and more adverse effects were observed among the patients with immunosuppressive regimens with no change in the rate of decrease in the eGFR (total number of infectious events in steroid treatment arm 182 vs. 111 in supportive care) [35]. However, of 309 patients who completed the 6-month supportive care run-in phase, 106 responded to supportive care (proteinuria level, <0.75 g of urinary protein excretion per day after the end of the run-in phase) and were not eligible for randomization. It needs to be highlighted that one third of patients were no longer suitable for randomization at the end of the run-in phase and the importance of RAS blockade was emphasized. The kidney function loss in the control group was 4 times slower in the STOP-IgAN trial than in the TESTING trial, suggesting a lower-risk population and/or differences in supportive therapy. Thus, it was assumed that low risk population of patients with IgAN with excellent prognosis with supportive care was selected in the STOP-IgAN trial. Nonetheless, detailed assessment of included patients with specific evaluation of histological renal findings and prolonged follow-up would be required for the elucidation of the results [35]. Included patients with inactive form of the disease might predominate in case of absent histological evaluation of renal specimens in the trials and the treatment of patients with inactive forms is useless. Recently, after ten years of follow-up from this study, the significant number of patients in both arms (supportive care plus immunosuppression and supportive care alone) reached the end-point with no benefits seen from the immunosuppression arm [36].

The enteric budesonide was evaluated for the treatment of IgAN in a European multicenter RCT [37]. In the NEFIGAN trial, a novel targeted-release formulation of budesonide was evaluated, designed to deliver the drug to the distal ileum with suspected suppression of B cells and inhibition of production of Gd-IgA1 moleculs transported to blood in patients with IgAN [37]. The results of the Nefigan study showed a significant reduction in proteinuria with full stabilization of eGFR without any serious side-effects [37]. A confirmatory phase 3 trial is currently underway (NCT 03643965). Other potential protease treatment with selective cleavage of IgA1 reverses mesangial deposits and hematuria in animal model and on human kidney biopsies [38,39].

Among patients with chronic kidney disease, regardless of the presence or absence of diabetes, the risk of a composite of a sustained decline in the estimated GFR of at least 50%, end-stage kidney disease, or death from renal or cardiovascular causes was significantly lower with dapagliflozin than with placebo. IgAN made up a significant proportion of the non-diabetic subgroup and the HR was an impressive 0.79 for IgAN. Unlike corticosteroid, the side effect profile is likely more favourable [40].

### 2.3. High Risk Patients with IgAN

Nevertheless, high risk patients with a rapid decrease in the GFR and crescentic glomerulonephritis should be treated in addition to supportive treatment with combined immunosuppression (corticosteroids and cyclophosphamide) in a regimen for induction treatment of ANCA vasculitides (Table 1) [16]. Extreme conditions with crescents involving >50% of glomeruli were considered by the KDIGO guidelines [16]. However, these cases are very rare. Just a few crescents are evaluated in majority of forms of crescentic IgAN, for which the need for aggressive treatment is doubtful. Moreover, a few glomeruli in biopsy specimens are sometimes detected with difficulties for calculation of a valid percentage of crescents. Furthermore, the percentage of glomeruli with crescents changes within a few days which makes comparison impossible among individual patients within cohort studies. Crescentic lesions were not found to have a prognostic value in the Oxford and VALIGA studies, but there was a bias in favour of using of corticosteroid/immunossupression treatment [4,41]. Another international study [7] demonstrated that C correlated with E1 and was associated with the use of CS/IS. The revised Oxford classification [42] suggested considering C1 (1–24% of glomeruli) and C2 (≥25% glomeruli with crescents). Crescents in 16% glomeruli increased the risk of renal function decline in untreated cases and crescents in 25% glomeruli predicted an unfavourable outcome independent of treatment.

Undoubtedly, crescents are a marker of histological activity but crescents can regress and do not require treatment in case of involvement of low percentages of glomeruli [43]. However in untreated patients, the negative effect of crescents on the renal function decline is well known [43]. Treatment with corticosteroids/immunossuppresion might to be initiated in case of involved crescents lesions >25% of the glomeruli (C2) and in patients with C1 involving >16% of glomeruli with other signs of disease activity such as endocapillary hypercellularity (E1 according to the Oxford classification) [43]. The importance of identifying patients at risk of progression was detailed mentioned and the task remains to avoid of exposure to immunosuppression regimen unnecessarily in patients with low risk of progression of renal function. The new international risk-prediction tool was recently developed in patients with IgAN [44,45].

Other immunosuppressive agents including calcineurin inhibitors, azathioprine, mycophenolate salts (MMF) or high-dose immunoglobulins failed to show an evident benefit or manifested with toxicity and therefore they were not recommended in clinical practice (Table 1) [46,47,48,49]. A Chinese trial randomized patients to 6 months of full dose steroids or lower dose steroid with MMF [50]. Complete proteinuria remission was similar between the two groups after one year but with fewer steroid-related adverse events in the arm with MMF [50]. Nevertheless, it was not a multiethnic study population, not all patients were treated by means of RAS-blockade and the time of follow-up was too short to evaluate the effect on renal parameters [50]. Although a possible positive effect of mycophenolate mofetil treatment in IgAN was noticed in a randomized controlled trial in China [51] with a significant reduction in the percentage of patients positive for histological change of E1 [50], the effect in Caucasian was not assessed [52] apart from the recent study on the effect of MMF therapy with a significant histological reduction in E1 score in repeated renal biopsies after 24 months (*p* < 0.0001) [53]. Further studies are needed for the assessment of treatment with mycophenolate mofetil in patients with IgAN. On the other hand several authors suggested potential benefits of rapamycin in animal and cell models of IgAN [54].

Moreover, Hydroxychloroquine in addition to optimized RAAS inhibition significantly reduced proteinuria in patients with IgAN over 6 months without evidence of adverse events. Undoubtadly, these findings require confirmation in larger treatment trials [55].

The question of the efficacy of fish oil in IgAN is uncertain (Table 1) [16]. Many randomizedclinical trials testing the efficacy of fish oil in patients with IgAN provided contrasting results [16]. It was reported that daily treatment with fish oil for 2 years may reduce the progression of renal function with few side effects in patients with IgAN [56]. Fish oil was also recommended by KDIGO guidelines if it was tolerated [16,34].

The role of adrenocorticotropic hormone (ACTH) was considered in the treatment of resistant glomerular diseases [57]. The potential involved mechanisms imagine stimulation of endogenous steroid production, activation of melanocortin receptors on inflammatory cells and direct binding to melanocortin receptors on the podocyte [57]. Administration of a 6-month course of ACTH gel in patients with IgAN at high risk of progression (proteinuria > 1 g per 24 h despite documented ACEI/ARB therapy and adequate blood pressure control for >3 months, 24-h creatinine clearance >30 mL/min/1.73 m^2^) was prospectively investigated in an open-label pilot study (NCT 02282930). A significant decline in 24-h urinary protein (2.6 to 1.3 g; *p* = 0.007) with no significant changes in eGFR (65.5 to 61.1 mL/min, *p* = 0.1) was detected at 12-month follow-up in patients with IgAN treated with 6 months of ACTH [58].

A systematic review and meta-analysis of 14 studies indicated that tonsillectomy may induce clinical remission and decrease the rates of ESRD in IgAN patients [59]. Another multicenter controlled trial did not show a beneficial effect of tonsillectomy combined with steroid pulse therapy over steroid pulses alone to increase the incidence of clinical remission [60]. In the large VALIGA cohort (the European validation study of the Oxford classification of IgAN) of 1.147 European subjects with IgAN, no significant correlation was found between tonsillectomy and renal function decline [61].

A therapeutic option targeting B-cell pathway treatment against the production of Gd-IgA1 and its specific antibodies, such as rituximab, was involved [62,63]. A multicenter trial of 34 adult patients wih biopsy-proven IgA nephropathy and proteinuria > 1 g per day, maintained on ACEIs or ARBs with well-controlled blood pressure and eGFR < 90 mL/min/1.73 m^2^, were randomized to receive supportive therapy either alone or with rituximab. Rituximab effectively depleted B cells (a monoclonal antiCD20 antibody) but neither serum levels of Gd-IgA1 nor its antibodies were reduced and the addition of rituximab failed to improve eGFR decline and proteinuria reduction [62]. CD20 positive B cells were targeted by rituximab but IgA positive plasma cells secreting antibodies were not affected therefore the treatment with rituximab was not efficient in patients with IgAN.

The activation of complement plays an important role in the pathogenesis of IgAN [64,65,66,67,68,69,70]. It was suggested that the process occurs both systemically on IgA-containing circulating immune complexes, and also locally in glomeruli, and is mediated through both the alternative and lectin pathways [13]. Pathway components were presented in the mesangial immunodeposits, including properdin and factor H (alternative pathway) and mannan-binding lectin, mannan-binding lectin-associated serine proteases 1 nad 2, and C4d (lectin pathway) [13]. Deletion of complement factor H-related genes 1 and 3 was identified as protective against the disease in GWAS [9,67].

C5a is a potent local inflammatory mediator and the presence of C5a in the kidney correlates with histological severity and proteinuria in IgAN [71]. Targeting C5a enables the suppression of local inflammation, contributing to progressive renal disease including the maintaining of the formation of C5b-9 (membrane attack complex), which plays a crucial role in the elimination of gram negative bacteria [71]. Avacopan (CCX 168), an inhibitor of the C5a receptor [72], was evaluated in an open-label Phase II trial in patients with IgAN. At the end of twelve weeks, proteinuria reduced in 6 of the 7 patients and a significant improvement of UPCR < 1 g/g was detected in 3 of the 7 patients. Undoubtedly, larger studies with longer follow up are needed in IgAN. Nevertheless, the efficacy of avacopan was confirmed in patients with ANCA-associated vasculitis where avacopan allowed the replacement of high-dose corticosteroids with respect of adverse effects of hepatic dysfunction and an increased risk of infection in a small amount of patients [73].

The alternative pathway forms an essential amplification mechanism for the activation of the classical and lectin pathways, resulting in enormous opsonisation and generation of the terminal lytic pathway [74]. The two proteases of Factor D and Factor B are elementary for this amplification process [74]. Selective reversible inhibitors of Factors B and D were developed to efficiently block the activation of alternative pathways [74]. A phase II trial of LNP023, a first in the class of oral inhibitor of Factor B, was recently recruited in patients with IgAN (Table 2). The key component of the lectin pathway demonstrates Mannose-binding lectin associated serine protease 2 (MASP-2), which elevates the production of C3 convertase and leads to further inflammatory consequences. Targeting MASP-2 seems to be promising in terms of reducing the activation of the glomerular lectin pathway and not affecting the formation of C3 convertase through the classical and alternative pathways. Presently, the MASP-2 inhibitor OMS 721 is being assessed in Phase II and Phase III studies in IgAN (Table 2).

The clinical effect of atacicept was investigated for treating systemic autoimmune diseases [75]. Studies in patients with IgA nephropathy investigate the contribution of dysregulation of IgA1 secretion in the intestinal epithelium to see if intestinal immunity or mucosal immunity inhibitors of overexpression of APRIL and B cell-activating factor would have a clinical effect [17,76,77]. Additional potential immunosuppressive regimens (such as blisibimod(NCT02062684)—A selective antagonist of the B-cell activating factor, atacicept (NCT02808429)—An inhibitor of B-cell activating factor (BLyS) and a proliferation-inducing ligand (APRIL), bortezomib—A proteasome inhibitor, fostamatinib–an inhibitor of spleen tyrosine kinase, leflunomide—A pyrimidine synthesis inhibitor) are currently being tested in ongoing trials (Table 2) [78,79,80,81,82,83].

BAFF (B-cell activating factor) and April (a proliferation inducing ligand) are members of the tumour necrosis factor family which mediate B-cell function and survival [84]. BAFF and April levels are elevated in the serum of patients with IgAN and correlate with the disease activity [85]. BAFF and April are bound by TACI (transmembrane activator and calcium-modulator and cyclophilin ligand interactor), which mediates their downflow effects through the NF-kB pathway. Blisibimod is a selective antagonist of BAFF and atacicept is a fusion protein containing the extracellular ligand binding domain of TACI and is able to block the downflow effects of BAFF and April.

The clinical effects of atacicept and blisibimod were investigated for treating systemic autoimmune diseases, including SLE and rheumatoid arthritis [75,86,87]. Studies in patients with IgAN investigate the contribution of dysregulation of IgA1 secretion in the intestinal epithelium to see if intestinal immunity or mucosal immunity inhibitors of overexpression of APRIL and B cell-activating factor would have a clinical effect [17,76,77].

Bortezomib, a plasma cell proteasome inhibitor, is applied in the treatment of multiple myeloma [88]. Proteasomes are indispensable intracellular protein complexes which destroy useless and impaired proteins by proteolysis [88]. Proteasomes are switched to immunoproteasomes but the dysregulation of the proteasome:immunoproteasome axis was confirmed in mononuclear cells in patients with IgAN with overexpression of the immunoproteasome, increased nuclear translocation of factors related to the NF-kB pathway, and more severe disease symptoms including higher proteinuria [89]. Nowadays, the treatment with bortezomib is more common in amyloidosis, lymphomas, tumours and antibody mediated allograft rejection [90]. A clinical trial to assess the safety and efficacy of bortezomib in patients with IgAN is currently underway (Table 2) but the use of bortezomib in mostly young asymptomatic patients with IgAN would probably be limited by the adverse effects of bortezomib (thrombocytopenia, rash, peripheral neuropathy, fatigue and anorexia) [90].

Tyrosine kinase pathways were asserted in the homeostasis of many diseases, and targeting of the tyrosine kinase signalling pathways was considered for treatment of immune-mediated glomerulonephritides [91]. Spleen tyrosine kinase is a non-receptor tyrosine kinase which might regulate the amount of key pathogenic pathways in IgAN [80]. Spleen tyrosine kinase is a signal transducer following B-cell receptor activation, arranging downflow signalling and promoting B-cell maturation and survival [80]. Spleen tyrosine kinase phosphorylation with the release of pro-inflammatory mediators was activated by stimulation of mesangial cells in vitro with IgA1 acquired from patients with IgAN [80].

Moreover, higher renal expression of spleen tyrosine kinase was confirmed in patients with endocapillary hypercellularity compared to patients without this sign in renal biopsy [92]. Fostamatinib, a selective inhibitor of spleen tyrosine kinase, was investigated in patients with rheumatoid artritis with a favourable effect on disease activity compared to placebo but with frequent adverse effects mostly involving diarrhoea and hypertension [93]. A Phase II trial of fostamatinib in patients with IgAN was recently completed.

Endothelin-1 (ET-1) is a growth factor for mesangial cells [94] and was shown to play a role in the progression of kidney disease in transgenic animals. In human subjects, urinary excretion of endothelin-1 correlates with the severity of kidney disease [95]. Expression of endothelin-1 and endothelin B receptor (mediating vasodilation and natriuresis), but not endothelin A receptor (mediating vasoconstriction and cell proliferation) was demonstrated in patients with IgAN and high-grade proteinuria [96] suggests that activation of the endothelin system in renal tubular cells may partly be a response to protein overload. We demonstrated association of the progresson of IgAN with polymorphisms of the ET-1 gene [97]. Association of ET-1 expression with the progression of IgAN was also confirmed using molecular profiling [98]. A specific ET-receptor antagonist (FR 139317) was shown to suppress the development of histologic lesions and proteinuria in ddY mice with IgAN [99]. Sparsentan, a dual inhibitor of the angiotensin II type 1 (AT1) and endothelin type A (ET-A) receptors was recently shown to significantly decrease proteinuria compared to irbesartan in patients with focal segmental glomerulosclerosis [100] and is currently tested in patients with FSGS in a phase 3 trial [101]. Recently, the PROTECT Study (Phase 3 NCT 03762850) evaluating the long-term nephroprotective potential of sparsentan for the treatment of IgAN was initiated. Activation of Nrf2/Keap 1 by bardoxolone methyl results in the suppression of the main proinflammatory transcription factor NFkappaB and activation of some antioxidative pathways [102]. In patients with type 2 diabetes and CKD4 bardoxolone, methyl was shown to increase the glomerular filtration rate by almost 50% [103]. Recently released data (PHOENIX–NCT03366337) demonstrated a significant increase in eGFR of 8 mL/min/1.73 m^2^ in patients with IgAN treated with bardoxolone [104]. The increase in eGFR is, however, associated with a proportional increase in albuminuria with uncertain impact on the long-term outcome of the patients [105]. The updated KDIGO guideline will soon be released [34] and the current recommendation from KDIGO came from 2012 prior to STOP-IgAN, TESTING, Nefigan and DAPA-CKD. This is a rapidly evolving field and hence the importance of this review on currently recruiting trials was emphasized.

## 3. Conclusions

**Figure 1:**
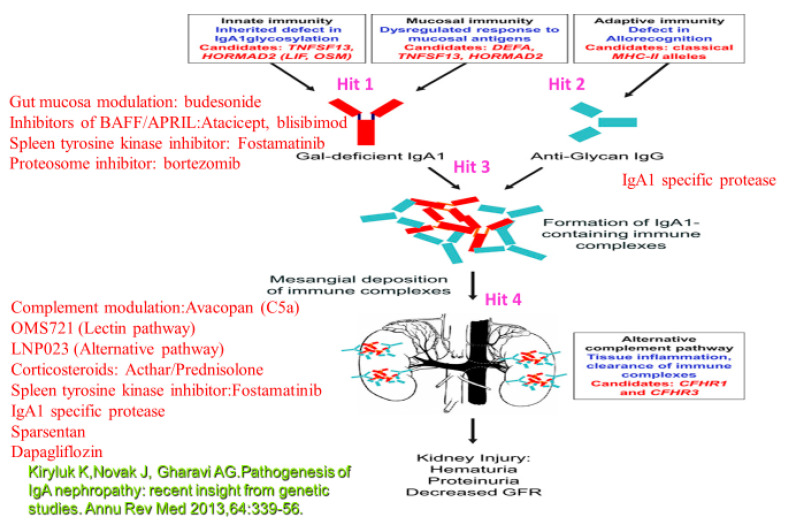
Therapeutic options related to the different targets. Target 1 **(Hit 1)**: B-lymphocyte activation results in the production of Gd-IgA1 (IgA1 poorly O-glycosylated at the hinge region). **Hit 2**: B-cell production of anti-Gd-IgA (IgG). Inhibitors of BAFF/APRIL:Atacicept, blisibimod; Spleen tyrosine kinase inhibitor: Fostamatinib;Gut mucosa modulation: budesonide; Proteosome inhibitor: bortezomib, microbiome modulation (may modulate B-lymphocyte activity with the reduction of Gd-IgA1). **Hit 3**: IgA1 specific protease. **Hit 4**:Spleen tyrosine kinase inhibitor:Fostamatinib; Corticosteroids: Acthar/Prednisolone, Complement mediation:Avacopan (C5a), OMS721 (Lectin pathway), LNP023 (Alternative pathway); IgA1 specific protease; Sparsentan; Dapagliflozin.

In conclusion, IgAN is a disease with variable clinical course. Validated urinary or serum biomarkers, able to provide information on the activity of the disease and the extent of fibrosis, are needed to stratify high risk patients with the necessity of use of immunossuppressive regimens with respect to possible adverse events. Nevertheless, IgAN patients with proteinuria < 1 g/d, and/or hypertension and a reduced GFR should obtain optimized supportive treatment with the inhibitors of the RAS. In IgAN patients with persistent proteinuria ≥ 1 g/d, despite 3–6 months of optimized supportive care (including RAS and blood pressure control), and GFR > 50 mL/min per 1.73 m^2^, a 6-month course of corticosteroid treatment is indicated. Combined immunosuppression with corticosteroids and cyclophosphamide is reserved for high risk patients with a rapid decrease in the GFR and crescentic glomerulonephritis. Nowadays, many specific biological regimens in RCT are evaluated with expected common use in the future (Table 2). The search for an effective and well tolerated treatment of IgAN, directly targeting its pathogenetic mechanisms, continues (Figure 1).

## Figures and Tables

**Table 1:** KDIGO guidelines for the treatment of IgAN [16].

Intervention	Recommendation	Grade
**Antiproteinuric and Antihypertensive Therapy**	Long-term treatment of ACE inhibitors or ARBs is recommended for patients with proteinuria > 1 g/day, with up-titration of the drug depending on blood pressure to achieve proteinuria < 1 g/day.	**1B**
	A target blood pressure of < 130/80 mmHg is recommended for patients with proteinuria < 1 g daily, and <125/75 for patients with proteinuria >1 g daily.	**Not graded**
**Corticosteroids**	A 6 month course of corticosteroids is recommended in patients with persistent proteinuria of >1 g/day despite 3–6 months of optimal supportive care and GFR > 50 mL/min per 1.73 m^2^.	**2C**
**Other Immunosuppressive Agents**	Patients with crescentic IgAN involving over 50% of glomeruli and rapidly progressive course should be treated with steroids and cyclophosphamide.	**2D**
	Not treating with corticosteroids combined with cyclophosphamide or azathioprine (unless crescentic forms with rapidly progressive course).	**2D**
	Not using immunosuppressive regimen in patients with GFR < 30 mL/min per 1.73 m^2^ (unless crescentic forms with rapidly progressive course).	**2C**
	Not using MMF.	**2C**
**Fish oils**	Fish oils may be potentially useful in patients with persistent proteinuria ≥ 1 g/day, despite 3–6 months of optimized supportive care.	**2D**
**Tonsilectomy, Antiplatelet Agents**	Not recommended.	**2C**

**Table 2:** Clinical trials in patients with IgA nephropathy—recruiting.

	Phase	Target	Clinical Trials in Patients with IgA Nephropathy—Recruiting	Estimated Enrollment				
				Number of pts	Inclusion Criteria	Treatment—Both Arms	Primary Endpoint	Time of Follow Up
Therapeutic options related to different targets of the pathogenesis (See Figure 1)								
NCT03633864	2	1	Fecal microbiota transplantation	30	eGFR:20–120 mL/min/1.73 m^2^, PU > 1 g/d	Exp:FMT arm/Biol.FMT	Change of urinary protein for 24 h	8 wks
NCT04438603	NA	4	The Role of T/B Cell Repertoire in Non-invasive Diagnosis and Disease Monitoring in pts with IgAN	50	eGFR ≥ 30 mL/min/1.73/m^2^	ACEI/ARB for non-progressive IgAN; Isu:CPA i.v./MMF p.o. for progressive IgAN	Urinary protein remission rate	2 yrs
NCT03001947	NA	others	Registration Initiative of High Quality (INSIGHT)	10,000	Biopsy proven IgAN	Observational/no intervention	Mortality, renal outcome (doubling of S-creat or ESRD)	10 yrs
NCT04042623	2	4	AVB-S6-500 (inhibitor of GAS6/AXL signaling pathway)	24	eGFR ≥ 45 mL/min/1.73 m^2^, PU 1–3 g/d	Experimental: Treatment with AVB-S6-500	Incidence od AE, UPE (g/d)	14 wks
NCT03188887	3	4	TIGER study (Treatment of IgA nEphropathy according to Renal lesions)	122	Biopsy proven IgAN < 45 days, PCR ratio > 0.75 g/g (within 30 days the renal biopsy)	RAS blockade treatment/Corticotherapy + RAS blockade treatment	Change of PCR, decline of eGFR	2 yrs
NCT03945318	1	1	BION-1301, a humanized IgG4 anti-a proliferation-inducing ligand (APRIL) monoclonal antibody	92	Biopsy proven IgAN within the past 10 years, PU ≥ 0.5 g/24 h, eGFR ≥4 5 mL/min/1.73 m^2^ or 30–45 mL/min/1.73 m^2^ with RB within 2 years before day 1	Bion-1301/placebo	Incidence and severity of Treatment Emergent Adverse Events (TEAEs)	2 yrs
NCT04014335	2	4	IONIS-FB-LRx, an inhibitor of complement factor B	10	Biopsy proven IgAN	IONIS-FB-LRx	Percent reduction in 24-h urine protein excretion	29 wks
NCT02954419	NA	others	IgA nephropathy biomarkers evaluation study (INTEREST)	2000	Biopsy proven IgAN	no intervention	A doubling of serum creatinine level from baseline, progression to ESRD, death	10 yrs
NCT03418779	2, 3	4	Treatment effects of chinese medicine with immunosuppression therapies	56	Biopsy proven IgAN within 6 months before enrollment, PU > 1 g/24 h, eGFR 20–60 mL/min/1.73 m^2^	herbal compound/Isu (Prednisolon, CPA)/optimized supportive care	Increase in GFR from the baseline at the 24th week and 48th week, ESRD	1 yr
NCT03373461	3	4	LNP023 (complement-factor-B-inhibitor)	146	eGFR ≥ 30 mL/min/1.73/m^2^, PU > 1 g/d, v accination against Neisseria meningitis	LNP023/Placebo	Change of UPCR at baseline and day 90	180 days
NCT03608033	3	4	OMS 721 (mannan-binding lectin serine protease 2 (MASP2) protein inhibitor)	450	PU > 1 g/d within 6 months prior to screening, eGFR ≥ 30 mL/min/1.73/m^2^	OMS 721/Placebo	Change from baseline in 24-h UPE in g/day at 36 weeks from beginning of treatment	3.5 yrs
NCT03643965	3	1	NefIgArd (Nefecon-budesonide modified-release capsules)	360	eGFR ≥ 35 mL/min/1.73/m^2^ and ≤90 mL/min/1.73/m^2^, PU >1 g/24 h or UPCR ≥ 0.8 g/g	Nefecon/Placebo	Change of UPCR at baseline and 9 months	25 mth
NCT03762850	3	4	PROTECT (Sparsentan-a dual inhibitor of AT1 and ETA receptor)	280	eGFR ≥ 30 mL/min/1.73/m^2^, PU > 1 g/24 h	Sparsentan/Irbesartan	Change of UPCR at baseline and week 36	110 wks

Abbreviation: wks—weeks, yrs—years, mth—months.
